# Endoscopic Retrograde Cholangiopancreatography Using a Dual-Lumen Endogastroscope for Patients with Billroth II Gastrectomy

**DOI:** 10.1155/2013/146867

**Published:** 2013-05-27

**Authors:** Wei Yao, Yonghui Huang, Hong Chang, Ke Li, Xuebiao Huang

**Affiliations:** Department of Gastroenterology, Peking University Third Hospital, 49 North Garden Road, Haidian District, Beijing 100191, China

## Abstract

*Objective*. To evaluate the safety and efficacy of a dual-lumen forward-viewing endoscope for ERCP in patients with prior Billroth II gastrectomy. *Methods*. The records of 46 patients treated with ERCP by a dual-lumen forward-viewing endoscope after Billroth II gastrectomy from 2007 to 2012 were reviewed. *Results*. The success rate of selective cannulation was 82.6% (38/46). Of the 38 cases with successful selective cannulation, endoscopic sphincterotomy was achieved in 23 cases by placing the needle knife through the 2nd lumen, while endoscopic papillary balloon dilatation was conducted in the other 15 cases. Of the 8 failed cases of selective cannulation, 6 had failed afferent loop intubation, and 3 of these 6 patients had Braun's anastomosis. The safety and efficacy of catheter-assisted endoscopic sphincterotomy were increased by placing the needle knife through the 2nd lumen without altering the conventional endoscopic sphincterotomy procedure. *Conclusions*. A dual-lumen forward-viewing endoscope can be safely and effectively used to perform ERCP in patients with a Billroth II gastrectomy, except for patients with additional Braun's anastomosis.

## 1. Introduction

Endoscopic retrograde cholangiopancreatography (ERCP) is commonly practiced for the diagnosis of biliary and pancreatic disorders, with advantages of minimal invasion, repeatable operation, and short recovery time [1]. However, it is difficult to perform ERCP in patients who have received Billroth II gastrectomies due to their reconstructed gastrointestinal anatomy. The orifice of the afferent loop is closed or hidden, while the length of afferent loop is elongated. The absence of gastric antrum support increases the difficulty of afferent loop endoscopic intubation. ERCP in patients after Billroth II gastrectomy is usually performed with a side-viewing or forward-viewing endoscope [1–4]. Both of the instruments have distinct advantages and drawbacks. The forward-viewing endoscope provides clear vision of the direction of the afferent loop but lacks an en face view of the papilla, thus resulting in a higher intubation rate than the side-viewing endoscope but in a lower cannulation rate [5]. On the other hand, the side-viewing endoscope provides an en face view of the papilla and provides an elevator, which makes the cannulation and placement of stents in biliary or pancreatic ducts easier. However, the risk of intestinal injuries and perforation may be increased in patients with the afferent loop positioned at a sharp angle due to the limited visual field of the side-viewing endoscope. Even though afferent loop intubation is successfully achieved, the following biliary cannulation is difficult for both types of endoscopes due to the reversed papilla position.

 The double-balloon endoscope, a type of one-lumen forward-viewing endoscope, has gained popularity recently, as it is associated with a high success rate of ERCP [6, 7], though it is not available nationwide in our country. The device features balloons that can be inflated during intubation, which enlarges the intestines to help locate the papillae. After locating the papillae, the operation of the double-balloon endoscope is the same as that of a regular forward-viewing endoscope. In other words, the difficulties of cannulation still exist. 

Fujita et al. inserted two instruments in one channel to successfully cannulate papillae in situ, which was deep-buried in the diverticulum of two patients after initial ERCP failed. Through the single channel of the duodenoscope, a forceps was delivered to grip and pull out the papilla from the diverticulum, and then a cannula was inserted to perform the cannulation [8]. Their report suggests that the cooperation of two instruments can facilitate papillary cannulation in cases with difficult anatomy. However, one channel provides limited space, which leads to mutual interference of two instruments and insufficient surgical view. A forward-viewing endoscope with double lumens may be a solution to this disadvantage, because it allows the delivery of two surgical instruments through different channels and thus solves the aforementioned problems. The dual-lumen forward-viewing endoscope is commonly used to remove polyps or gastric cancer. However, to authors' knowledge, the application of dual-lumen endoscope to ERCP has not been reported yet. The current study reported the outcome of dual-lumen endoscope used in ERCP for patients with history of Billroth II gastrectomy. The purpose of this study is to access the safety and feasibility of dual-lumen endoscopes in ERCP for this particular group of patients. 

## 2. Patients and Methods 

This retrospective study was approved by the institutional review board in our hospital. We reviewed the patients treated with ERCP from 2007 to 2012. The patients without Billroth II gastrectomy history or operation by dual-lumen endoscope were excluded. A total of 46 patients were selected. After signing the consent forms, the chart of these patients was reviewed from the time they were administered in the hospital to the time they were discharged. 

### 2.1. ERCP Procedures

**Figure 1:**
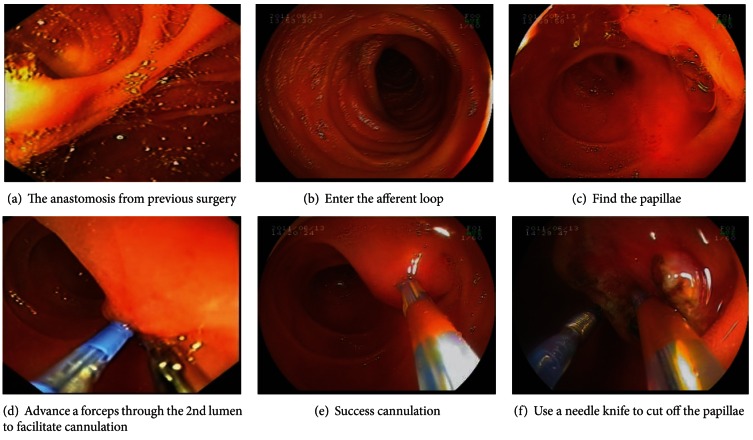
Images of the key procedures using a double-lumen forward-viewing endoscope for ERCP with prior Billroth II gastrectomy.

The ERCP surgery was conducted by senior surgeons. In this study, a successful ERCP was defined as an achievement of selective cannulation. A forward-viewing dual-lumen gastroscope (Olympus GIF-2T100) was used. The endoscope has a diameter of 12.6 mm and a length of 103 cm. The endoscope was not equipped with a lift clamp (elevator). The channel with a 3.7 mm diameter (the 1st lumen) was mainly used for ERCP, while the channel with a 2.8 mm diameter (the 2nd lumen) was used for auxiliary function. Due to the previous anastomosis (Figure 1(a)), the afferent loop and efferent loop of patients were distinguished through the following methods: (1) reviewing the previous surgery records: if the large curve was proximal for patients in the left-lateral supine position, the inferior orifice indicated the afferent loop; and vice versa; (2) observing the intestinal cavity: it is easier for the scope to enter the efferent loop and the afferent loop usually has bubbles and bile; and (3) using the C-arm fluoroscope: the path of the endoscope can be visualized under fluoroscopic guidance. If the endoscope is positioned in the right upper quadrant of the stomach, most of the time it enters the afferent loop and vice versa. The position of the patients was frequently changed until the scope entered the afferent loop.

The procedure is illustrated in Figures 1(b)–1(f). Briefly, the endoscope was placed through the afferent loop and the papilla was located (Figure 1(b)). After the duodenal papilla was visualized (Figure 1(c)), a papillotome was advanced through the 1st lumen to perform selective cannulation. An Allis forceps was placed through the 2nd lumen to hold the papilla to assist in cannulation if needed (Figures 1(d) and 1(e)). Following selective cannulation, endoscopic sphincterotomy (EST), endoscopic nose-bile drainage (ENBD), endoscopic retrograde biliary drainage (ERBD), endoscopic papillary balloon dilatation (EPBD), and/or common bile duct stone removal were performed. During EST, a needle knife can be placed via the 2nd lumen to cut the papilla along the indwelling cholangiography catheter or a sphincterotome can be delivered through the 1st lumen (Figure 1(f)). 

## 3. Results 

The reviewed patients were 29 males and 17 females, with an average age of 68.2 years (range, 38 to 87 years). The clinical history showed that Billroth II gastrectomies were performed for gastric cancer in 15 patients and for peptic ulcer management in 31 patients. Surgery types included antecolic gastrojejunostomy in 18 patients and retrocolic gastrojejunostomy in 13 patients, while the other 15 patients had no definite surgery history (Table 1). In addition, 5 patients received Braun's anastomosis and 9 patients had other biliary surgeries. The time after Billroth II gastrectomy ranged from 1 to 48 years. Before ERCP, all patients received a B-mode ultrasound examination and/or magnetic resonance cholangiopancreatography angiography (MRCP). The results showed varying degrees of choledochectasia with a mean diameter of 1.4 cm (range, 0.9–2.3 cm). Of the 46 cases, 5 cases were diagnosed with common bile duct malignant obstruction, 38 cases had common bile duct stones, and 3 cases had Duodeno papillary benign stenosis (Table 1).

Out of 46 patients, ERCP was successful in 38 patients, resulting in a success rate of 82.6%. Of the 8 failed cases, failure of afferent loop intubation occurred in 6 cases and unsuccessful selective cannulation in 2 cases. Of the 5 cases who previously received Braun's anastomosis, afferent loop intubation was unsuccessful in 3 cases, resulting in 60% failure rate. Of the 38 successful ERCP cases, 16 cases were assisted with the Allis forceps passed through the 2nd lumen to hold the papilla to facilitate selective cannulation. Following successful ERCP, EST was performed in 23 patients, and endoscopic papillary balloon dilatation (EPBD) was performed in 15 patients. Thereafter, bile duct stones were successfully removed in 31 cases, a biliary metallic stent was implanted in 4 cases of malignant obstruction, and ENBD was performed in 3 cases of Duodeno papillary benign stenosis. All patients recovered well, but 23 patients had a transient elevation of amylase level. Serious complications, such as bleeding, perforation, or pancreatitis, did not occur. 

## 4. Discussion 

The present study investigated the feasibility of using a dual-lumen forward-viewing endoscope for ERCP in patients who have received a Billroth II gastrectomy. The difficulty of performing ERCP after Billroth II gastrectomy results from the changed gastrointestinal anatomy, including the hidden afferent loop opening, the sharp-angulated afferent loop entrance, the elongated afferent loop, and the upside-down papilla. The forward-viewing endoscope obtains better views of the afferent loop traveling direction than the view of papillae location, which results in lower successful rate of cannulation. It has been shown that the success rate of papillae cannulation increases with the angle of endoscope from anterior-posterior direction, with 92.3% in forward-viewing endoscope [9], 94.7% in forward-oblique endoscope [10], and 98% in side-viewing endoscope [11]. The immediate replacement of forward-viewing endoscope with side-viewing endoscope is suggested when the cannulation is unsuccessful during ERCP [4]. 

 The dual-lumen forward-viewing endoscope has the advantages of forward-viewing endoscope and resolves the insufficient en face view of the papilla with permission of extra surgical tools through the additional lumen. There are not any specific difficulties in approaching the papillae with two instruments because the two channel tubes are basically parallel. Combined with the pulling of the gastrointestinal tract, the relative positions of the two instruments become complicated once their distance from the papillae is increased. Therefore, it is suggested to keep the end of the two lumens of forward-view scope within a relatively close distance from the papillae. 

In the present study, 16 out of 38 successful selective cannulation cases were achieved with an Allis forceps placed through the 2nd lumen to hold the papilla. The success rate of selective cannulation after afferent loop intubation was 95% (38/40), which is comparable to other series using side-viewing endoscopes [3, 12, 13]. The failure rate of endoscopic afferent loop intubation was 13% (6/46), of which 3 patients had a prior Braun's anastomosis. The failure rate of ERCP was 60% (3/5). In patients with Braun's anastomosis, the failure rate of ERCP is reported to be 67–70% [5, 14]. The difficulty of afferent loop intubation results from the elongated afferent loop and the increased number of anastomoses. To overcome these anatomical challenges, the use of a stiffer endoscope along with manual compression has been suggested to avoid loop formation at the ligament of Treitz and increase the success rate of afferent intubation [5]. The lower success rate of afferent loop intubation in the present series may be due to insufficient stiffness of the endoscope. Instruments, such as a polypectomy snare, can be placed into the endoscope to increase the overall stiffness during intubation. Alternative methods, such as a DBE or single-balloon endoscope can also improve the success rate in patients with Braun's anastomosis [15, 16].

To efficiently extract bile stones, it is recommended to perform EST before EPBD. However, because of the altered anatomy, EST can be dangerous and inadequate in patients with a Billroth II gastrectomy. One of the standard procedures of EST following ERCP in patients with a Billroth II gastrectomy is to rotate the sphincterotome to cut the reversed papilla. Another method is to use the needle knife to cut the papilla along the plastic stent previously placed in the common bile duct. Both methods are technically demanding in patients with a Billroth II gastrectomy and are associated with an increased risk of intestinal bleeding and perforation with an incidence reported to be as high as 10.2% [14]. As a result, some endoscopists suggest performing EPBD without EST to remove bile stones using endoscopes with special specifications [10, 17]. Both EST and EPBD were individually performed in the present series using a dual-lumen forward-viewing endoscope, and the safety and efficacy of catheter-assisted EST were increased by placing the needle knife through the 2nd lumen without altering the conventional EST procedures. 

In conclusion, our results suggest that a dual-lumen forward-viewing endoscope can be safely and effectively used to perform ERCP in patients with a Billroth II gastrectomy, except for patients with additional Braun's anastomosis.

## Figures and Tables

**Table 1:** Demographic and clinical characteristics of the recruited patients (*n* = 46).

Reasons of Billroth II gastrectomy
Gastric cancer (*n* = 15)
Peptic ulcer (*n* = 31)
Types of Billroth II gastrectomy
Antecolic gastrojejunostomy (*n* = 18)
Retrocolic gastrojejunostomy (*n* = 13)
Unknown (*n* = 15)
Diagnosis for ERCP
Common bile duct malignant obstruction (*n* = 5)
Common bile duct stones (*n* = 38)
Duodenopapillary benign stenosis (*n* = 3)
